# Prevalence and Associated Factors of Common Mental Disorders Among Adult Residents in Silte Zone, Southern Ethiopia

**DOI:** 10.2174/1745017902117010128

**Published:** 2021-10-15

**Authors:** Mohammed Muze, Mehbub Denur, Mubarek Hussein, Mufta Muzemil, Mubarek Yesse, Shemsu Kedir

**Affiliations:** 1 Department of Nursing, College of Medicine and Health Sciences, Werabe University, Southern Ethiopia; 2 Department of psychiatry, Werabe Comprehensive Specialized Hospital, Southern Ethiopia; 3 Department of Quality Assurance, Werabe Comprehensive Specialized Hospital, Southern Ethiopia; 4 Department of Public Health, Collage of Medicine and Health Sciences, Werabe University, Southern Ethiopia

**Keywords:** Common mental disorders, Self-reporting questionnaire, Social support, Anxiety, Somatic, Symptoms, Depression

## Abstract

**Introduction:**

Mental health problems appear to be increasing in importance in Africa. Mental and substance use disorders were the leading cause of yearly lived with disability in Sub-Saharan Africa. Evidence from previous studies shows considerable variation in the prevalence of these disorders. The most acceptable explanation for this wide variation is likely to be the fact that socio cultural factors are major determinants of mental disorders. Therefore a mental disorder has to be understood in a specific setting to develop effective and tailored interventions.

**Objective:**

The objective of this study was to determine the prevalence and associated factors of common mental disorders among adult residents in Silte Zone, southern Ethiopia

**Methods:**

Community based cross-sectional study was conducted in the study area. A total of 1178 adults were selected by using a three-stage systematic sampling technique. The Self-Reporting Questionnaire (SRQ-20) was used to determine the prevalence of common mental disorders. Data were analyzed by using SPSS version 20. Both bivariate and multiple logistic regression analyses were employed to identify factors associated with common mental disorders.

**Results:**

The prevalence of common mental disorders among adults found to be 39.7%. Increased age (OR = 1.114; 95% CI = 1.095, 1.134), being female (OR = 9.421; 95% CI = 5.947, 14.926), poor social support (OR = 1.987; 95% CI = 1.358, 2.907) and having life threatening experience (OR = 2.162; 95% CI = 1.825, 2.562) were significantly associated with common mental disorders.

**Conclusion:**

In the study, the magnitude of common mental disorders remains high in the study area. Increased age, being female, poor social support and having life-threatening experience were significantly associated with common mental disorders.

## 1 INTRODUCTION

Common mental disorders are a group of distress states manifesting with anxiety, depressive and unexplained somatic symptoms that typically come up in the community and primary care setting and also frequently occur together with a shifting combination of symptoms over time indicating emotional or mental abnormality [1, 2] Mental disorders have been recognized as the most public health important globally, around 450 million people currently suffer from such conditions, placing mental disorders among the leading causes of ill-health and disability worldwide [3].

The global prevalence of common mental disorders from 1980–2013 was approximately 1 in 5 people experienced during the 12-month preceding assessment [4]. Adult mental disorders are found to be associated with high role impairment and economic burden [5, 6]. According to the World Economic Forum, mental illness will account for more than half of the economic burden of disease over the next two decades – more than cancer, diabetes and chronic respiratory diseases combined. About 54% of the economic burden of disease falls to low- and middle-income countries [7]. Mental health problems appear to be increasing in importance in Africa. Mental and substance use disorders were the leading cause of YLDs (yearly lived with disability) in Sub-Saharan Africa [8].

Evidence from previous studies shows that mental disorders are common in all countries, there is a considerable variation in the prevalence of these disorders. In developed worlds, the past 12-month prevalence of mental disorders was found 14% in Greece [9] and 44% in a review of Bangladesh, India, and Pakistan [10]. In Africa, the past 12-month prevalence of common mental disorders was found between 10.3%- 33.6% [11-14]. This can be explained by deference in sociocultural factors across countries. Mental problems are influenced by social factors such as gender, social class, race and ethnicity, household patterns, poverty, violence, and other stressful social environments. They are not unique to any part of the globe [15, 16].

Myths and misconceptions about mental illness contribute to the stigma, which leads many people to be ashamed and prevents them from seeking help. Generally, people who have mental disorders are considered lazy, unintelligent, worthless, stupid, unsafe to be with, violent, always in need of supervision, possessed by demons, recipients of divine punishment, unpredictable, unreliable, irresponsible, without conscious, incompetent to marry and raise children, unable to work, affect rich people, increasingly unwell throughout life, and in need of hospitalization [17].

Researchers found that Khat use is directly linked to common mental disorders [18]. Khat is a flowering plant that contains the alkaloid cathinone, which is said to cause excitement [19], which is common in Horne of Africa in including study area. According to the 2016 Ethiopia Demographic and Health Survey report, 12% of women and 27% of men reported the use of Khat [20]. The presence of khat in the study area may increase the burden of common mental disorders.

The majority of the global burden of mental disorders is located in low and middle-income countries [21]. Therefore, integration of mental health in primary health care in resource-limited settings is more important for two reasons. The first reason is mental health resources are unlikely to be adequate to address the burden of mental disorders in low and middle-income countries. The second reason is most patients with mental disorders, particularly less severe disorders, prefer to seek care from their own family or primary health care settings [22]. Evidence from previous studies shows that mental disorders are common in all countries, there is a considerable variation in the prevalence of these disorders. Apart from methodological factors, the most acceptable explanation for this wide variation is likely to be the fact that socio-cultural factors are major determinants of mental disorders. Therefore a mental disorder has to be understood in a specific setting to develop effective and tailored interventions. Hence, this community-based study was intended to determine the prevalence and associated factors of common mental disorders among adult residents in the study area.

## 2 METHODS

### 2.1 Study Design, Period and Area

Community-based cross-sectional study was conducted from January to April/2019 in Silte Zone. Silte zone is located 172km south of Addis Ababa, the capital of Ethiopia. The zone is divided into 10 Weredas (Districts) and 3 administrative towns. Wereda and administrative town are a cluster of kebeles which are the smallest unit administration. Ten Weredas (Districts) and 3 administrative towns consist of a total of 204 kebeles, and 211,012 households. According to the 2007 central statistical agency of Ethiopia, the estimated population size of the zone is 1,033,954, and 584,184 were adults.

### 2.2 Study Subjects

The source population of this study was all adults. Individuals whose age >=18 yrs and who have lived in the study area for at least 6 months were included. Individuals who had a hearing problem, cognitive and memory impairment were excluded.

### 2.3 Sample Size Determination

The sample size was determined by using single population proportion formula: with an assumption of prevalence rate of 33.6% (11) for common mental disorders from a previous study in Jimma southwest of Ethiopia, which gave the maximum sample size than prevalence rates found elsewhere in the country, margin of error(d) 4%, design effect of 2, Z=1.96 at 95% confidence level. Using the above information, the calculated sample size was 1071, and after adding a 10% potential non-response rate, the final sample size was 1179.

### 2.4 Sampling Technique

A three-stage sampling technique was employed for the study. The study unit was households with the assumption that each household would have a study subject. Using a simple random sampling technique, in the first stage, 4 Weredas were selected from the 13 Weredas of the zone. In the second stage, 36 kebeles were randomly selected out of 121 kebeles of four Weredas. The calculated sample size was proportionally allocated to selected kebeles based on the number of households found in the kebeles. In the third stage, 1179 households were selected by using a systematic random sampling technique. In the selected kebeles, there were 34187 households. When more than one study subject were found in one household, a lottery method was used to select a participant.

### 2.5 Measurements and Data Collection Tools

Twelve data collectors collected the data by using paper-based structured interviewer-administered questionnaire. The questionnaire was adapted from articles published in peer-reviewed journals. A Self-reporting questionnaire (SRQ-20) was used to classify whether a common mental disorder was present or not. Each of the 20 items scored 0 or 1. A score of 1 indicates the presence of symptoms, and 0 indicates the absence of symptoms during the last month. The maximum score is, therefore, 20. It has been validated in Ethiopia [23]. A score above the cut-off point indicates the existence of a probable mental disorder. In neighboring country Eritrea, a study found a good sensitivity and specificity at a lower cut of value (5/6) for SRQ-20 [24]. Therefore, in this study, 6 was used as a cut-off value to determine the presence or absence of CMDs. Another study in another part of Ethiopia also used 6 as a cut-off value for SRQ-20 [25]. The Oslo-3 Social Support Scale (OSS-3) was used to collect social support characteristics. The scale consists of three items. The sum score ranges from 3 to 14. By adding the three items, those with 3–8 values were considered to have poor social support, while those with 9–11 and 12–14 values were considered to have intermediate and strong social support, respectively [26]. OSS-3 has acceptable internal consistency and construct validity in the general population [27]. Finally, stressful life events were assessed by using an adapted version of the List of threatening experiences (LTE) questionnaire. It is a 12 items instrument measuring common life events that tend to be threatening [28].

### 2.6 Data Quality Management

Trained data collectors collected the data. A locally validated and translated tool was used to collect the data. The instrument was pretested in 5% of the sample size. It was conducted on individuals’ study population who were not a part of the actual study. Based on the pretest results, the instrument was modified. Variables such as IV drug use, ethnicity, religion were modified in the final tool. The principal investigators and supervisors checked out the collected data for completeness, accuracy, consistency and clarity every day and made amendments before the next data collection measure. Principal investigators carefully cleaned and entered collected data into a computer.

### 2.7 Data Processing and Analysis

Data were checked for completeness and consistencies, then edited, coded and entered by using Epi-Info version 7 and exported to SPSS version 20. Both bivariate and multiple logistic regression analyses were employed to identify factors associated with CMDs. Then all variables significantly associated with bivariate analysis were a candidate to multivariable logistic regression analysis. Multivariable analysis was performed to see an association between dependent and independent variables. To estimate the strength of association, an adjusted odds ratio (AOR) with a 95% confidence interval was reported between study variables. A P-value less than 0.05 is considered statistically significant.

## 3 RESULTS

### 3.1 Socio-demographic Characteristics of Respondents

Table **1** summarizes the socio-demographic characteristics of the study participants. Of the total 1179 initially planned for the study, 1168 participated in the study, with a response rate of 99%. The majority (63.7%) of the respondents were female. The majority of the participants were in the age group 45-54 years with a mean and standard deviation of 45±15 years. Most (91.8%) of the respondents were rural dwellers. Nearly two-third (74.7%) of study subjects had no formal education, and more than half (60%) of them were housewives. The average household size was 2±1.

### 3.2 Psycho-social and Lifestyle Factors

Among study participants, 530 (45.4%) reported no stressful life events, 463 (39.6%) reported one up to two stressful life events and the remaining 175 (15%) reported three and above stressful life events in the last four weeks. The majority (70.1%) of the participants had poor social support, 29.9% of the participants had intermediate social support and none of the study participants had strong social support. Out of the total 1168 study subjects, 876 (75%) never have a history of khat use, 235 (20%) reported the use of khat sometimes, and the rest 57 (5%) reported khat use usually. About 40% of the respondents had a family history of khat use. All of the respondents reported they are not current alcohol drinkers. Most (94.5%) of the respondents were non smokers.

### 3.3 Clinical Related Factors

About 582 (49.8%) of the respondents had a family history of mental illness, and 177 (15%) had a personal history of mental illness. Out of the total, 57 (4.9%) of the respondents had diabetes mellitus, 85 (7.5%) had hypertension and 68 (5.8%) had Asthma.

### 3.4 Prevalence of Common Mental Disorders

The prevalence of common mental disorders among adults was found to be 39.7% (N = 464/1168). When we look closely at the prevalence of specific symptoms of CMD (Table **2**), the following symptoms were found to be highly prevalent: headaches (44.8%), uncomfortable feelings in the stomach (35.1%), easily tired (44.7%), sleep badly (29.8%) and feel nervous, tense or worried (29.6%), whereas symptoms like difficult of enjoying in daily activities (4.9%), difficult in decision making in day to day life (4.9%), unable to play a useful part in life (4.9%), lost interest in things (9.8%), feel that you are a worthless person (9.9%) and trouble thinking (10%) were relatively less common (Table **2**).

### 3.5 Factors Associated with Common Mental Disorders

Both bivariable and multivariable logistic regression analyses were done. Initially, all variables included in the study were analyzed using bivariable logistic regression. Then variables with p-value <0.25 were included in the multivariable analysis (Table **3**). In the bivariable model, increased age, being female, low educational status, poor social support and having a life-threatening experience were positively associated with CMD; and all these associations were statistically significant. In the multivariable model older age (OR = 1.114; 95% CI = 1.095, 1.134), being female (OR = 9.421; 95% CI = 5.947, 14.926), poor social support (OR = 1.987; 95% CI = 1.358, 2.907) and having a life-threatening experience (OR = 2.162; 95% CI = 1.825, 2.562) were significantly associated with CMDs (Table **3**).

## 4 DISCUSSION

The aim of this study was to assess the prevalence of common mental disorders and their associated factors. The results showed that 39.7% of the adults had common mental disorders. Our result is comparable with a study carried out in another area of Ethiopia and a review done in three countries of Europe (Bangladesh, India, and Pakistan), where it was estimated at 33.6% [11] and 44% [10], respectively. On the other hand, our finding is higher than those of two area studies of Ethiopia, namely in Harari 14.9% (25), a review in Ethiopia 21.58% [29]. Our finding is also higher compared to two studies conducted in Kenya, and a study in Greece reported 10.3% [13], 10.8% [12] and 14% [9], respectively. The variation might be due to distinctions in the measurement tools, and the socio-culture distinctions between Ethiopia and the other counties.

Regarding factors, we found a highly increased prevalence in women compared to men; CMDs were 9 times higher among female respondents compared to males. This finding is supported by other studies [11, 30]. It may be attributed to gender-specific risk factors for common mental disorders that disproportionately affect women including gender-based violence, socioeconomic disadvantage, low income and income inequality, low or subordinate social status and rank and unremitting responsibility for the care of others [31]

Older age is also positively associated with CMDs, older age increases the likelihood of CMDs. This result is consistent with that of previous studies done in Ethiopia and Kenya [11, 25, 32]. There may be multiple risk factors for mental health problems at any point in life. Older people may experience life stressors common to all people; stressors may be more common in later life, like a significant ongoing loss in capacities and a decline in functional ability lead to common mental disorders.

Adults who had poor social support were 2 times more likely to develop CMDs compared to those who had good social support. Evidence showed that the impact of social support on mental health can occur through two mechanisms: either as a main effect influence in which social support has a beneficial effect on mental health regardless of whether or not the individuals are under stress, or social support improves the wellbeing of those under stress by acting as a buffer or moderator of that stress [33].

In the current study, a strong association between stressful life events and common mental disorders was observed. Stressful life event increases the likelihood of having common mental disorders. This could be explained by hormones, neuroendocrine mediators, peptides, and neurotransmitters involved in the body's response to stress. Many disorders originate from severe and prolonged stress that leads to mental disorders [34, 35]

### 4.1 Strengths and Limitations of the Study

The strengths of the study are the random sample of households, large sample size and the high response rate. Limitations of the study are the use of cross-sectional study design; we cannot permit conclusions about some variables, for example, about deciding whether common mental disorder symptoms are at risk or a consequence. The measurement used to diagnose common mental disorders was self-reported, which is subjected to recall and reporting bias. Further research should be conducted on risk factors for CMDs to strengthen and broaden our results.

## CONCLUSION

In the study, the magnitude of the common mental disorder remains high in the study area. Older age, being female, increased family size, poor social support, and stressful life event were significantly associated with common mental disorders. Based on our findings, we would like to forward the following recommendation; mental health screening and counseling should be strengthened in health facilities; due attention should be given to the mental health aspect of those older adults, women, adults who have poor social support. Future research could beneficially include consideration of the severity and impairment of activities of daily living caused by CMD and would contribute to an understanding of the impact of CMDs on the lives of this population.

## Figures and Tables

**Table 1:** Characteristics of adult residents in Silte zone, Southern Ethiopia, 2019, (n=1168).

**Variables**	**Categories**	**Frequency**	**Percent**
Age of respondent	18-24	172	14.7
	25-34	180	15.4
	35-44	136	11.6
	45-54	338	28.9
	55-64	235	20.1
	>=65	107	9.2
	Total	1168	100.0
Sex	Male	424	36.3
	Female	744	63.7
	Total	1168	100.0
Residency	rural	1072	91.8
	urban	96	8.2
	Total	1168	100.0
Marital status	married	736	63.0
	single	120	10.3
	separated	22	1.9
	widowed	290	24.8
	Total	1168	100.0
Educational status	Not read and write	873	74.7
	Primary	236	20.2
	Secondary	59	5.1
	Total	1168	100.0
Occupation	No job	59	5.1
	Housewife	702	60.1
	Student	119	10.2
	Farmer	288	24.7
	Total	1168	100.0
Family size	2-4	700	59.9
	5-7	374	32.0
	>=8	94	8.0
	Total	1168	100.0
Monthly income in Ethiopian birr	<400	189	16.2
	401-800	451	38.6
	801-1500	417	35.7
	>1501	111	9.5
	Total	1168	100.0

**Table 2:** Prevalence of common mental disorders and their symptoms among adult residents in silte zone, Southern Ethiopia, 2019.

**Variables**	**Response**	**Frequency**	**Percent**
Do you often have a headache?	no	645	55.2
	yes	523	44.8
	Total	1168	100.0
Is your appetite poor?	no	876	75.0
	yes	292	25.0
	Total	1168	100.0
Do you sleep badly?	no	820	70.2
	yes	348	29.8
	Total	1168	100.0
Are you easily frightened?	no	993	85.0
	yes	175	15.0
	Total	1168	100.0
Do your hands shake?	no	993	85.0
	yes	175	15.0
	Total	1168	100.0
Do you feel nervous, tense or worried?	no	822	70.4
	yes	346	29.6
	Total	1168	100.0
Is your digestion poor?	no	879	75.3
	yes	289	24.7
	Total	1168	100.0
Do you have trouble thinking clearly?	no	1050	89.9
	yes	118	10.1
	Total	1168	100.0
Are you unhappy?	no	994	85.1
	yes	174	14.9
	Total	1168	100.0
Do you cry more than usual?	no	1168	100.0
Do you find it difficult to enjoy your daily activities?	no	1111	95.1
	yes	57	4.9
	Total	1168	100.0
Do you find it difficult in decision making in day-to-day life?	no	1111	95.1
	yes	57	4.9
	Total	1168	100.0
Is your daily work suffering?	no	995	85.2
	yes	173	14.8
	Total	1168	100.0
Are you unable to play a useful part in life?	no	1111	95.1
	yes	57	4.9
	Total	1168	100.0
Have you lost interest in things?	no	1053	90.2
	yes	115	9.8
	Total	1168	100.0
Do you feel that you are a worthless person?	no	1052	90.1
	yes	116	9.9
	Total	1168	100.0
Has the thought of ending your life been on your mind?	no	995	85.2
	yes	173	14.8
	Total	1168	100.0
Do you feel tired all the time?	no	879	75.3
	yes	289	24.7
	Total	1168	100.0
Do you have uncomfortable feelings in your stomach?	no	758	64.9
	yes	410	35.1
	Total	1168	100.0
Are you easily tired?	no	646	55.3
	yes	522	44.7
	Total	1168	100.0

**Table 3:** Bivariable and multivariable logistic regression analysis of determinant factors for common mental illnesses among adult residents in Silte Zone, southern Ethiopia, 2019.

**Variables**	**Categories**	**CMDs**		**Crude odds ratio (95% C.I)**				**Adjusted odds ratio (95% C.I)**			
		**No**	**Yes**	**P value**	**COR**	**Lower**	**Upper**	**P value**	**COR**	**Lower**	**Upper**
Sex	Male	297	127	-	-	-	-	-	-	-	-
	Female	407	337	.000	1.936	1.503	2.494	.000	9.421	5.947	14.926
Education	Illiterate	468	405	-	-	-	-	-	-	-	-
	Literate	236	59	.000	.289	.211	.396	.160	2.121	0.148	3.916
Social support	Good	234	115	-	-	-	-	-	-	-	-
	Poor	470	349	.002	1.511	1.162	1.965	.000	1.98	1.358	2.907
LTE	As increased by one			.000	1.573	1.430	1.730	.000	2.16	1.825	2.562
Age	As increased by one			.000	1.056	1.047	1.066	.000	1.11	1.095	1.134

LTE refers Life-Threatening Experience

## Data Availability

The data supporting the finding of the article is available in the Zenodo Repository at zenodo.org, reference number https://zenodo.org/record/5536246#.YVRi7JpBzIU and DOI is 10.5281/zenodo.5536246.
